# Analysis of the Real Performance of Crumb-Rubber-Modified Asphalt Mixtures

**DOI:** 10.3390/ma15238366

**Published:** 2022-11-24

**Authors:** Francisco Javier Sierra-Carrillo de Albornoz, Fernando Moreno-Navarro, María del Carmen Rubio-Gámez

**Affiliations:** 1 Consejería de Infraestructuras, Fomento y Ordenación del Territorio Junta de Andalucía, Avda. Joaquina Eguaras 2, 18071 Granada, Spain; 2 Laboratory of Construction Engineering, University of Granada (LabIC.UGR), Avda. Severo Ochoa s/n, 18071 Granada, Spain

**Keywords:** asphalt, crumb rubber, cracking, stiffness, field performance

## Abstract

The main goal of this study is to evaluate the field performance of crumb-rubber-modified asphalt mixtures used as a surface layer on high-volume traffic roads. For this purpose, several road sections were constructed under different climate conditions and using control mixtures (manufactured with traditional SBS polymer-modified binders) and crumb-rubber-modified mixtures. After the construction of the different road sections, cores were taken at different periods of their service life (up to 63 months) and they were tested in the laboratory in order to assess the evolution of the density, stiffness and fatigue resistance of the layers. Based on the results obtained from tests, it can be concluded that under real severe traffic and climate conditions, asphalt mixtures manufactured with crumb-rubber-modified bitumen offer ageing and mechanical performance very similar to that offered by asphalt mixtures manufactured with traditional SBS-modified bitumen. Based on these considerations, this application can be an interesting solution to minimize environmental problems caused by end-of-life tires in landfills.

## 1. Introduction

End-of-life tires are one of the most widely produced types of solid waste around the world [1]. They are composed of high-quality materials such as rubber, metallic fibers, nylon fiber, etc., that are difficult to degrade but which can offer interesting solutions to primary materials when they are re-used for a second purpose. Therefore, during the last few decades, many efforts have been made to find applications for the valorization of end-of-life tires [2,3,4,5,6]. One of the most common and successful applications is to reuse their rubber as a binder modifier in bituminous mixtures. For this purpose, the rubber from the tire is transformed into crumb, and it is incorporated into the asphalt binder (wet process) or directly into the mixture as a part of the mineral skeleton (dry process) [7].

Its application as a modifier in bituminous mixtures permits the recovery of this waste in huge quantities, helping to contribute to circular economy principles (around 1000 tires could be reused per kilometer of road, when using a bituminous mixture modified with 0.5% of its total weight and a thickness layer of 6 cm). In addition, many laboratory studies have demonstrated that the use of crumb rubber as a modifier of asphalt mixtures improves their resistance to fatigue cracking [8,9] and to plastic deformation [10], reduces their ageing [11], increases their durability [12,13] or minimizes the rolling noise [14,15]. Thus, this method of valorization can result in more durable road pavements, with less need for maintenance, which contributes to the more sustainable and effective management of natural, economic and energy resources.

After decades of use, there are many experiences that have already proven the advantages of the application of crumb rubber from end-of-life tires as a bituminous mixture modifier [16,17,18,19,20,21,22,23,24]. However, in other countries, such as Spain [25,26], there are doubts about the efficiency in the long term, especially when they are applied on surface layers, on high-volume traffic roads or in severe climate conditions. Traditionally, the performance of the applications has not been properly documented and there is a lack of information, which prevents road administrators from selecting these materials instead of traditional SBS-modified binders (which have decades of success and trustable use). Therefore, the application of crumb rubber is not common under these circumstances. In addition, there is some skepticism about its workability in cold climates [27], its stability to form an homogeneous blend [8] or its skid resistance [28], as well as about its economic and environmental viability [29].

In order to determine in depth the real performance of crumb-rubber-modified asphalt mixtures, during the last few years, the road administration of the government of Andalusia (Spain) has applied them on the surface layers of some highway sections that experience high volumes of traffic and unfavorable climate conditions. To evaluate these materials, a study on the evolution of the mechanical performance of them during their service life in comparison to traditional SBS-modified material has been carried out in collaboration with the Laboratory of Construction Engineering of the University of Granada. For this purpose, cores directly extracted from the surface layer of the pavement at different dates were tested at the laboratory, determining the evolution of important parameters such as density, stiffness or fatigue cracking resistance. This paper summarizes the main results obtained in this research work so far.

## 2. Materials and Methods

### 2.1. Materials

This paper is focused on the study of 3 sections of the high-traffic-capacity road network of Andalusia (Spain), on the A-316 highway (Jaen) and A-92 highway (Granada, in two different locations). The main characteristics of these highway sections are summarized in Table 1. The A-316 offered less severe service conditions, with less than 600 heavy vehicles daily and no presence of snow/ice over the road surface. On the opposite site, the A-92-I offered the most severe conditions in terms of traffic and climate (more than 3000 heavy vehicles pass every day and the presence of snow, frost and chemical substances for de-icing is quite common for 5 months every year).

All these sections were constructed using a surface layer of 3 cm thickness and using different job mix formulas of BBTM 11B PMB 45/80-60 C (an asphalt mixture composed of a gap-graded mineral skeleton with a maximum aggregate size of 11 mm and manufactured with a crumb-rubber-modified asphalt binder [30]). In all the highway sections studied, a sub-section of around 400 m was also constructed using BBTM 11B PMB 45/80-60, manufactured with the same mineral skeleton but using a conventional SBS polymer-modified bitumen from a refinery (these reference sections were constructed side by side to ensure the same environmental and traffic conditions as the crumb-rubber-modified asphalt layers). Table 2 summarizes the main characteristics of the job mix formulas used in the construction of the different sections studied. As can be observed, all the job mix formulas offered similar properties (optimal binder content, density and voids) and mechanical performance, measured in laboratory tests conducted on mixture samples obtained in the asphalt plants used during the construction of the highway sections studied. In addition, it must be highlighted that there were no significant differences between them in terms of costs, workability or bitumen homogeneity.

**Figure 1:**
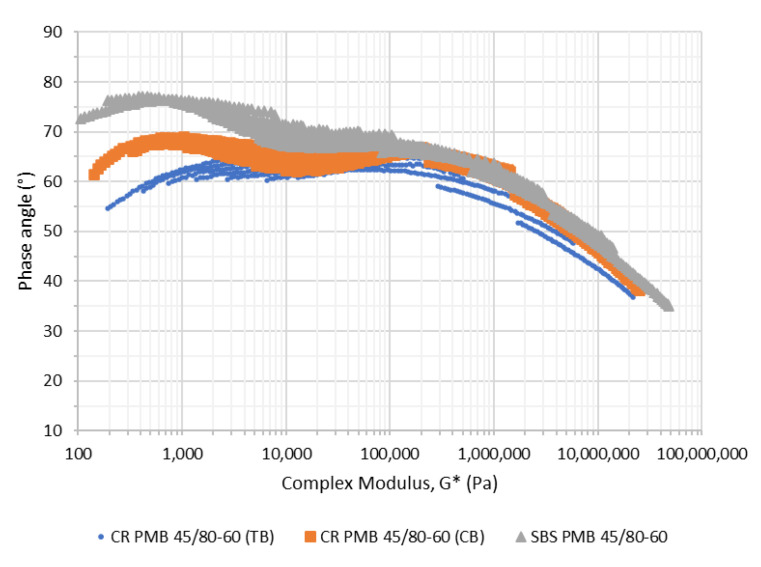
Rheological performance of the binders used in the manufacture of the mixtures.

Figure 1 shows the rheological properties (black diagrams) of the fresh binders used in the manufacture of the mixtures.

### 2.2. Testing Plan

**Figure 2:**
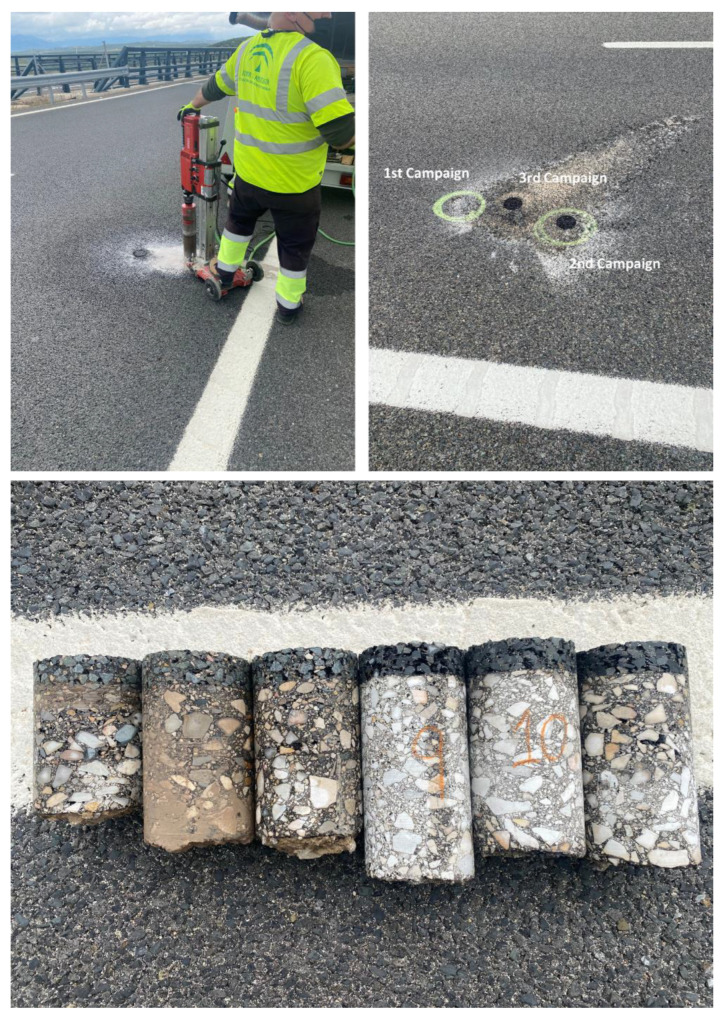
Details of the core extraction process.

During the service life of the three highway sections studied, cores from the surface layers were extracted at different times (Table 3). A total of 6 cores on each extraction campaign were obtained from the different mixtures studied. All these cores were obtained in the right carriageway over the wheel path at the same 6 different kilometric points in order to avoid the inclusion of new variables in the study (Figure 2). Once the cores were extracted, the 3 cm surface layers were sawed at the laboratory to be tested.

**Figure 3:**
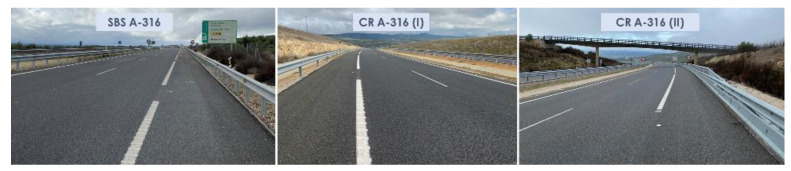
Aspects of the surface asphalt layers manufactured with SBS and crumb-rubber-modified binders on A-316 highway after 63 months of service life.

**Figure 4:**
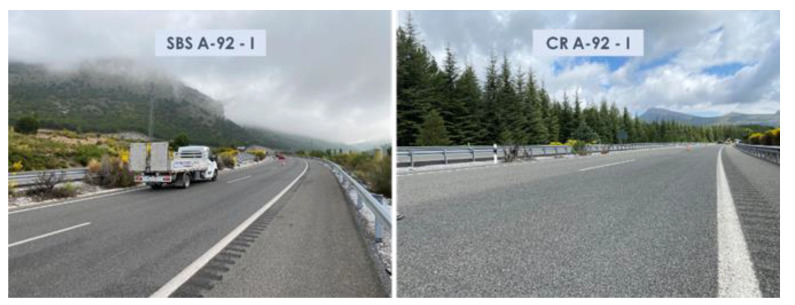
Aspects of the surface asphalt layers manufactured with SBS and crumb-rubber-modified binders on A-92 highway after 46 months of service life.

**Figure 5:**
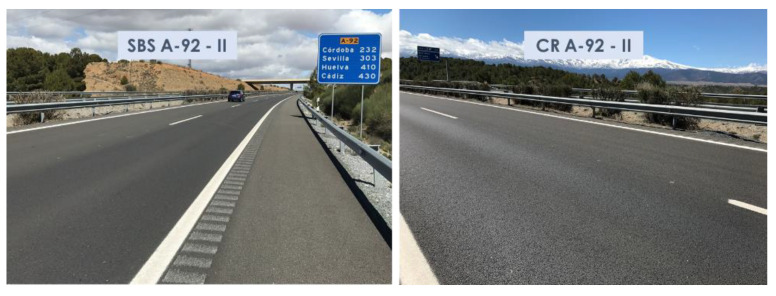
Aspects of the surface asphalt layers manufactured with SBS and crumb-rubber-modified binders on A-92 highway after 36 months of service life.

The superficial aspects of the highway sections studied during the last campaign are shown in Figure 3, Figure 4 and Figure 5.

**Figure 6:**
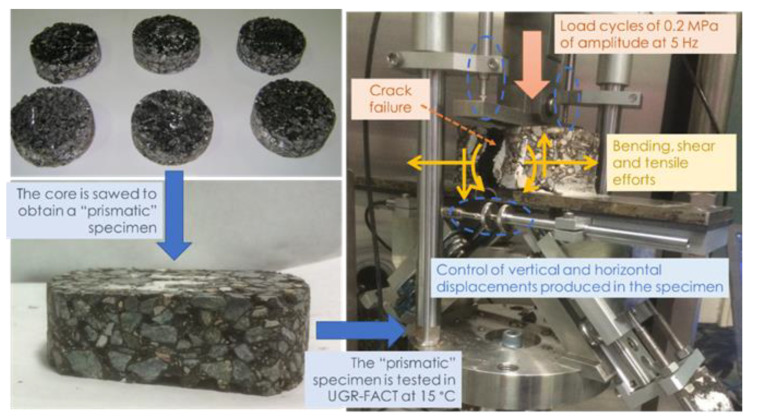
Schema of the UGR-FACT method.

Once the cylindrical cores were obtained from the road on the different dates, their apparent density (according to UNE-EN 12697-6 [31] for specimens with more than 10% of voids in mixture) was determined using the geometric procedure (where the mass of the core is divided by the volume of the core defined by their height and diameter). After this, their stiffness at 20 °C was measured according to UNE-EN 12697-26 (annex C) [36], where 15 indirect tensile load pulses (10 of conditioning, and the last 5 used for measured the average stiffness of the core), in the form of versine and with a 3 s duration for each one, were applied in two different diameters of the core (the value of stiffness is considered as the average of the average values obtained in the last 5 load pulses of each diameter). After this, the fatigue cracking resistance of the cores was measured using the University of Granada-Fatigue Asphalt Cracking Test (UGR-FACT) [37]. The configuration of the testing device [38] induced combined efforts in the cores (bending, tensile and shear), similar to those that occurred during their service life in the pavement layer (Figure 6). Based on the displacements measured and on the number of load cycles required to produce their failure (the total propagation of a crack causing the breakage of the specimen into two pieces), the fatigue resistance of the materials was obtained. This test was demonstrated to be a useful and sensitive tool to measure the influence of different design parameters, such as the aggregate nature [39], the type of binder [40,41,42], the use of additives [43] or the traffic and climatic service conditions [44,45], on the fatigue and structural resistance of asphalt mixtures. In addition, UGR-FACT was already demonstrated to be a good tool for studying the effect of sustainable technologies on asphalt materials’ cracking resistance, such as healing [46], different warm-mix asphalt technologies [47] or the addition of crumb rubber [9]. During this research, the cores obtained on different dates in the three highway sections were tested using versine load cycles of 0.2 MPa amplitude at a frequency of 5 Hz at 15 °C. For this purpose, they were sawed to obtain specimens with 100 mm maximum length, 60 mm maximum width and 30 mm thickness (Figure 6).

## 3. Analysis of Results

**Figure 7:**
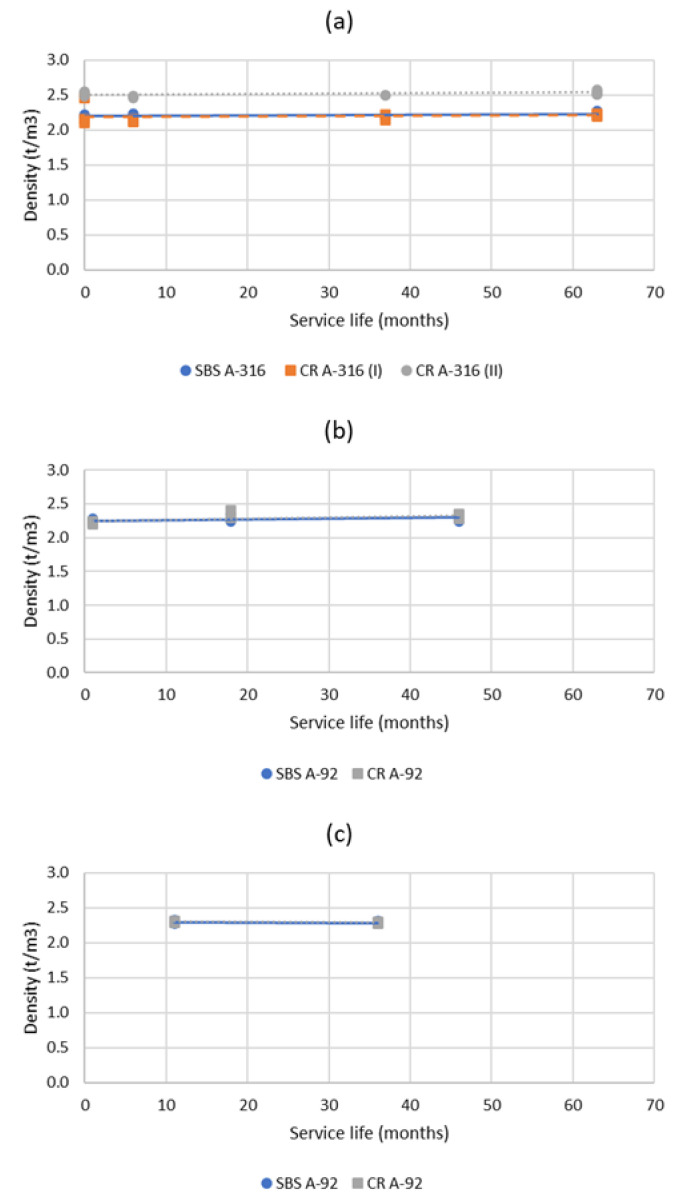
Evolution of the densities measured in the cores obtained along the service life of the highway sections studied: (**a**) A-316; (**b**) A-92-I; (**c**) A-92-II.

Figure 7 shows the evolution of the cores’ densities as a function of the service life, for each section of highway studied. As can be observed, slight variations were observed in terms of density of the asphalt layers along their service life (thus, the changes observed in their mechanical performance cannot be related to this parameter). In addition, it can be said that no differences were observed between the mixtures manufactured with the crumb-rubber-modified bitumen and those using the SBS-modified one (the evolution of the density of the layers is the same regardless the type of modified binder used). Furthermore, the binder content measured in the cores and their aggregate gradations were also very constant along the service life.

**Figure 8:**
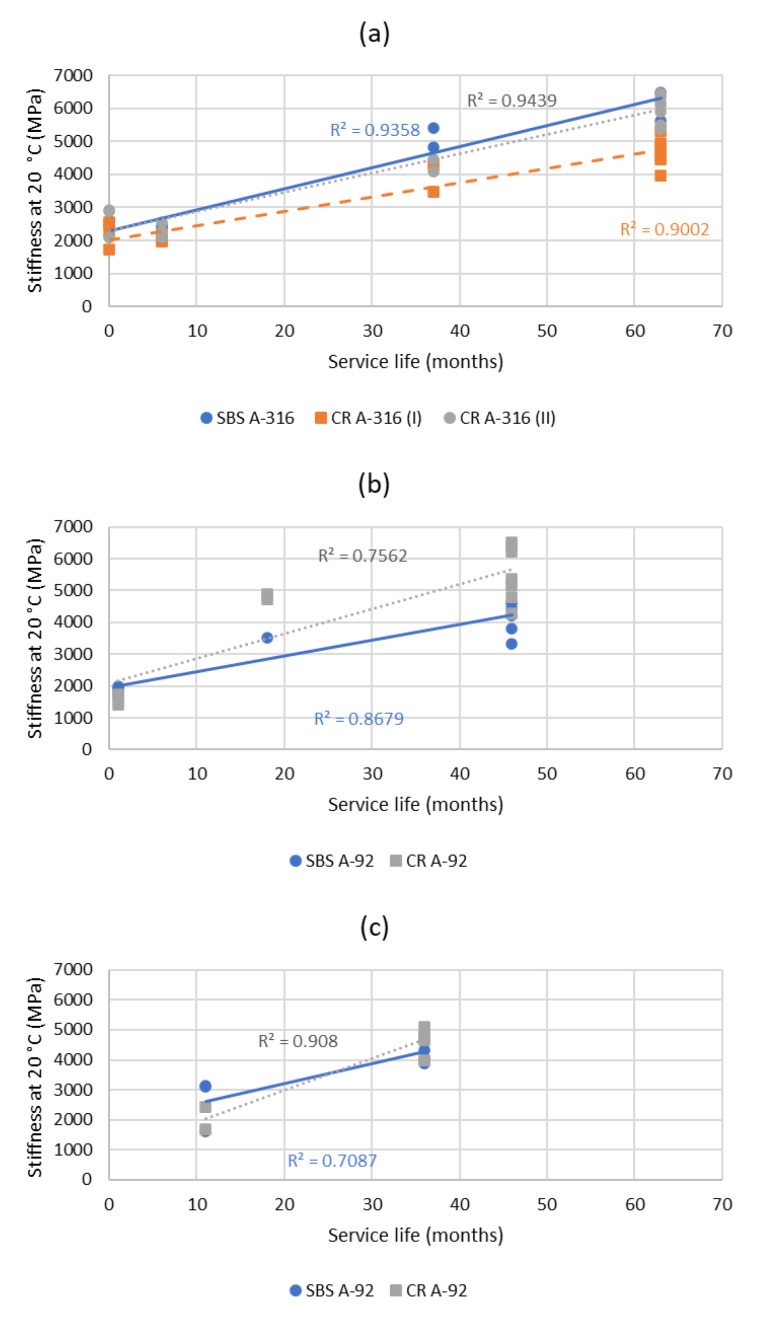
Evolution of the stiffness measured in the cores obtained along the service life of the highway sections studied: (**a**) A-316; (**b**) A-92-I; (**c**) A-92-II.

The evolution of the stiffness of the layers as a function of the service life is shown in Figure 8. It is observed that, due to ageing, the asphalt layers increased in stiffness linearly along their service life, regardless of the type of bituminous binder used (it must be highlighted the high correlation coefficient of the straight line). SBS polymer-modified asphalt mixtures had a rate of stiffness increment of around 64 MPa/month in the A-316 section, 49 MPa/month in the A-92-I section and 67 MPa/month in the A-92-II section (which gives an average of 60 MPa/month, with a coefficient of variation of 15%). Refinery crumb-rubber-modified asphalt mixtures (terminal blend) had a rate of stiffness increment of 58 MPa/month in the A-316 section, 78 MPa/month in the A-92-I section and 105 MPa/month in the A-92-II section (which gives an average of 80 MPa/month, with a coefficient of variation of 30%). Based on these results, it can be said that no significant differences were found between highway sections and that the crumb-rubber-modified asphalt binder suffered a 25% higher stiffness variation than the SBS-modified one. Nonetheless, when analyzing the mixture manufactured with the continuous-blend crumb-rubber-modified bitumen in the A-316 section, it was observed that the rate of stiffness increment was 43 MPa/month, which was 30% less than the stiffness increment observed in the SBS-modified one for the same circumstances. Therefore, it can be concluded that crumb-rubber-modified bitumens and SBS-modified bitumens offer stiffness effects in a similar order during their service life.

**Figure 9:**
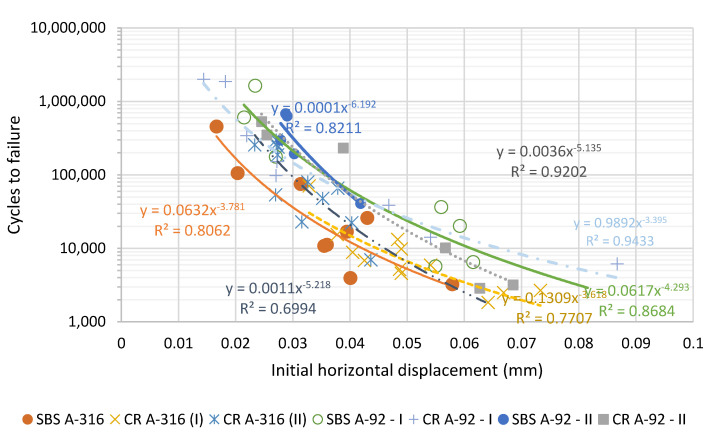
Fatigue laws measured in the cores obtained along the service life of the highway sections studied.

Figure 9 shows the fatigue laws obtained from the cores extracted at different periods of the service life of the highway section studied using UGR-FACT. As can be observed, there were no significant differences between the mixtures manufactured with the crumb-rubber-modified binders and the SBS-modified ones (for the same job mix formula, no significant differences have been obtained). Instead, it is shown that the BBTM 11B job mix formula used in A-92 offered slightly higher fatigue resistance than those used in A-316. Thus, it can be said that crumb-rubber-modified asphalt binders also offer similar long-term performance under cycling fatigue loads as the SBS-modified bitumens.

**Figure 10:**
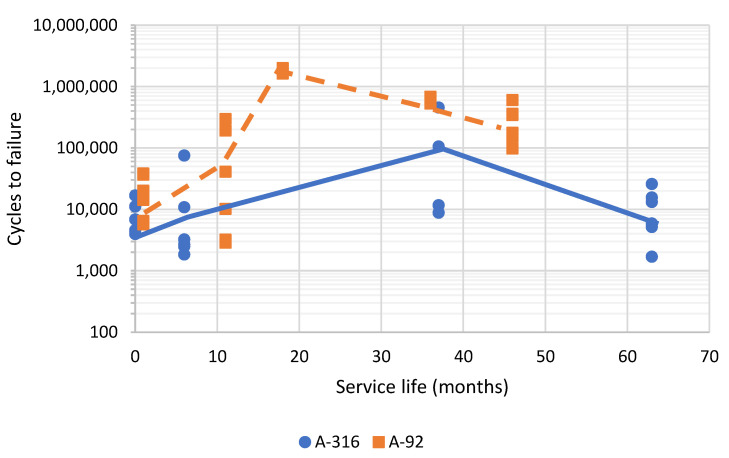
Evolution of the resistance to fatigue cracking of the highway section studied as a function of the service life.

Figure 10 shows the evolution of the fatigue cracking resistance of the asphalt layers studied as a function of the service life in A-316 and A-92 sections, measured using UGR-FACT. Due to the similarity in the fatigue laws obtained, the results were grouped in order to show the trend marked by the service life. In this respect, it was observed that the fatigue resistance of asphalt layers tends to increase during the first periods of their service life (until 20–40 months, depending on the traffic volume and due to the strain hardening suffered [9]), and after this, it starts to decrease, which marks a point at which the traffic loads would start to deteriorate the asphalt layer.

## 4. Conclusions

This paper summarizes the results obtained in a research study whose main objective was to carry out an in-depth analysis of the mechanical performance of crumb-rubber-modified bitumen under severe traffic and climate conditions in real road pavements. For this purpose, cores were extracted on different dates from the surface layers of three highway sections that were constructed using a crumb-rubber-modified bitumen (PMB 45/80-60 C), and they were compared with cores extracted on the same date and at the same location from surface layers constructed with SBS-modified bitumen (PMB 45/80-60). These cores were tested at the laboratory to determine their density (UNE-EN 12697-6), stiffness (UNE-EN 12697-26, annex C) and resistance to fatigue (UGR-FACT). From the results obtained in this study, the following conclusions can be drawn:

- The density of the surface layers studied was not affected by the service life (being constant over time and very similar to those reached after the pavement’s construction), regardless of the type of asphalt binder used (SBS or crumb-rubber-modified).
- The stiffness of the surface layers studied was significantly affected by the service life due to the effects of the environmental agents. The mixture BBTM 11B showed a rate of stiffness increment between 43 and 105 MPa per month, but we did not observe a clear dependence of this rate on the type of binder used or the climate conditions in the highway section studied. Thus, it can be concluded that surface asphalt mixtures manufactured with SBS or crumb-rubber-modified binders offer a similar stiffening process during their service life.
- The results obtained in terms of long-term resistance (fatigue cracking) also demonstrated that the mixtures manufactured with SBS or crumb-rubber-modified binders offer similar performance. In both types of materials and in the three highway sections studied, it was observed that during the first 20–40 months of service life (depending on the volume of traffic supported), the surface asphalt mixtures increased in resistance to fatigue loading (due to the strain hardening phenomenon), and after this period, they became susceptible to fatigue damage (which, in the end, would cause the failure of the layer).

The results obtained in the study presented show that, under real severe traffic and climate conditions, the asphalt mixtures manufactured with crumb-rubber-modified bitumen offer ageing and mechanical performance very similar to that offered by asphalt mixtures manufactured with traditional high-performance SBS-modified bitumen. As future research, it would be interesting to continue this study by adding aspects related to the serviceability of pavements and road safety, such as IRI or skid resistance, and applying a life cycle cost analysis to determine the materials’ real efficiency.

## Figures and Tables

**Table 1:** Characteristics of the service conditions in the sections studied.

	A-316	A-92-I	A-92–II
Date of traffic opening	November, 2015	September, 2017	July, 2018
Annual average daily traffic (Number of vehicles)	8000	18,000	11,000
Percentage of heavy traffic (over the total number of vehicles)	8	17	7
Climate conditions	~750 m above sea level; rarely frost/snow over the road surface during autumn/winter; maximum average temperatures in summer ~36 °C; minimum average temperatures in winter ~4 °C	~1400 m above sea level; very frequent frost/snow over the road surface during autumn/winter; maximum average temperatures in summer ~30 °C; minimum average temperatures in winter ~1 °C	~1100 m above sea level; frequent frost/snow over the road surface during autumn/winter; maximum average temperatures in summer ~33 °C; minimum average temperatures in winter ~4 °C;

**Table 2:** Characteristics of the job mix formulas used in the construction of the highway sections studied.

	SBS A-316	CR A-316 (I)	CR A-316 (II)	SBS A-92	CR A-92
Type of aggregate coarse fraction (6/12 mm)	Ophite	Trachyte	Ophite	Ophite	Ophite
Type of aggregate fine fraction (0/6 mm)	Siliceous	Limestone	Siliceous	Limestone	Limestone
Type of filler	Portland Cement	Portland Cement	Portland Cement	Portland Cement	Portland Cement
Type of binder	SBS PMB 45/80-60	CR PMB 45/80-60 Continuous blend (CB)	CR PMB 45/80-60 Terminal blend (TB)	SBS PMB 45/80-60	CR PMB 45/80-60 Terminal blend (TB)
Optimal binder content (%/mixture weight)	4.78	4.79	4.82	4.80	4.75
Aparent density (g/cm^3^), UNE-EN 12697-6 [31]	2.386	2.087	2.391	2.146	2.178
Voids in mixture (%), UNE-EN 12697-8 [32]	14.3	16.9	15.1	16.5	18.0
Dry indirect tensile strength (kPa) [33]	1702	1670	1691	1287	1100
Indirect tensile strength ratio (%) [34]	88.9	94.8	84.3	91.9	95.5
Wheel tracking slope (mm/10^3^ cycles) [35]	0.06	0.04	0.08	0.06	0.05

**Table 3:** Dates on which the cores were extracted in each highway section studied.

Core Extraction Campaign	A-316	A-92-I	A-92-II
1	0 months (November, 2015)	1 month (October, 2017)	11 months (June 2019)
2	6 months (May, 2015)	18 months (March, 2019)	36 months (July 2021)
3	37 months (January, 2019)	46 months (July, 2021)	-
4	63 months (February, 2021)	-	-

## Data Availability

Not applicable.
